# Differentiating Late Awakeners from Non-Awakeners in Comatose Cardiac Arrest Survivors: Diagnostic Value of Multimodal Monitoring in Patients with Indeterminate Prognosis

**DOI:** 10.3390/diagnostics16040558

**Published:** 2026-02-13

**Authors:** Hyo Joon Kim, Sang Hoon Oh, Jee Yong Lim

**Affiliations:** 1 Department of Emergency Medicine, Seoul St. Mary’s Hospital, The Catholic University of Korea, Seoul 06591, Republic of Korea; liebestest@hanmail.net (H.J.K.); mdshow@hanmail.net (S.H.O.); 2 International Healthcare Center, Seoul St. Mary’s Hospital, The Catholic University of Korea, Seoul 06591, Republic of Korea

**Keywords:** cardiac arrest, neuron-specific enolase, delayed awakening

## Abstract

**Background**: Current guidelines recommend prognostication at 72 h after cardiac arrest, yet a subset of patients (Late Awakeners) recover consciousness after this window. This study investigated diagnostic markers to distinguish Late Awakeners from those with permanent poor outcomes (Non-Awakeners) to prevent premature withdrawal of life-sustaining therapy. **Methods**: We analyzed adult OHCA patients treated with TTM from 2009 to 2019 who remained comatose (Glasgow Coma Scale Motor score < 6) at 72 h. Patients were categorized as Late Awakeners (obeyed commands > 72 h) or Non-Awakeners. The diagnostic performance of maximal Neuron-Specific Enolase (NSE) levels within 72 h and brainstem reflexes was assessed using receiver operating characteristic (ROC) analysis. Model calibration was evaluated using the Hosmer–Lemeshow test, and internal validation was performed using bootstrap resampling. **Results**: Of 213 patients comatose at 72 h, 20 (9.4%) were identified as Late Awakeners. The median time to awakening was 4.4 days (IQR 3.4–8.3) from ROSC. Late Awakeners exhibited significantly preserved corneal reflexes (85.0% vs. 20.2%) compared to Non-Awakeners. The optimal NSE cut-off value to predict late awakening was <89.5 ng/mL (Sensitivity 95.0%, Specificity 50.3%, AUC 0.801). A multimodal approach combining NSE < 90 ng/mL and preserved corneal reflexes achieved a high specificity of 93.2% and an AUC of 0.899 (optimism-corrected: 0.896) for predicting late recovery. At six-month follow-up, 74.3% of Late Awakeners achieved good neurological outcome (CPC 1–2). **Conclusions**: Approximately 9% of patients comatose at 72 h eventually regain consciousness with favorable long-term outcomes. A multimodal diagnostic model combining intermediate NSE thresholds and preserved brainstem reflexes can effectively identify these Late Awakeners, suggesting that observation should be extended for patients fitting this profile.

## 1. Introduction

Out-of-hospital cardiac arrest (OHCA) remains a major global public health challenge, accounting for substantial mortality and long-term disability [1]. Despite the implementation of the Chain of Survival and advances in post-resuscitation care, the rate of survival with good neurological function is still unsatisfactory. The widespread adoption of targeted temperature management (TTM) has marked a paradigm shift in post-cardiac arrest care, significantly mitigating reperfusion injury and suppressing cerebral metabolic demand [2,3]. However, as survival rates have improved, clinicians are increasingly confronted with the challenge of hypoxic-ischemic brain injury (HIBI), which remains the leading cause of death among hospitalized survivors. In this era of TTM, accurate neuroprognostication has become the cornerstone of critical care, as it directly informs decisions regarding the withdrawal of life-sustaining treatment (WLST) and the optimization of intensive care resources [4]. However, the timing and criteria for WLST vary substantially across countries, healthcare systems, and individual clinical contexts [5]. In regions where WLST decisions are made relatively early, there is a heightened risk that potentially salvageable patients may not be given sufficient time to demonstrate neurological recovery. Conversely, in settings where aggressive treatment is continued for prolonged periods, the identification of patients who are unlikely to recover becomes critical for appropriate resource allocation and family counseling. These regional and institutional differences in end-of-life practices underscore the need for objective diagnostic markers that can guide decision-making independent of local WLST culture.

Current international guidelines, including those issued by the European Resuscitation Council (ERC) and the American Heart Association (AHA), advocate for a multimodal prognostication strategy [6,7]. This approach integrates clinical examination, electrophysiological studies, neuroimaging, and serum biomarkers to predict neurological outcomes. The guidelines universally recommend delaying the prognostication assessment until at least 72 h after the return of spontaneous circulation (ROSC) to minimize the confounding effects of therapeutic hypothermia and sedation. The rationale is to ensure that the patient’s lack of responsiveness is due to permanent brain injury rather than reversible metabolic suppression.

However, in real-world clinical practice, strict adherence to this 72 h window often proves insufficient for a distinct subset of patients. The pharmacokinetics of sedative and analgesic agents, such as midazolam and fentanyl, can be highly unpredictable in post-arrest patients [8]. The metabolic clearance of these drugs is frequently delayed due to TTM-induced hypothermia and concurrent organ dysfunction, such as acute kidney injury (AKI) or hepatic impairment, which are common complications of post-cardiac arrest syndrome (PCAS) [9]. Consequently, a phenomenon known as late awakening has been increasingly recognized, wherein patients remain comatose at the standard 72 h assessment point but regain consciousness days or even weeks later [10,11].

These Late Awakeners represent a critical blind spot in current prognostication algorithms. While recent literature suggests that Late Awakeners constitute a non-negligible proportion of the comatose population, they are often at high risk of being misclassified as having a poor prognosis. If clinicians strictly adhere to the absence of a motor response at 72 h as a criterion for poor outcome, there is a substantial risk of a self-fulfilling prophecy, leading to the premature withdrawal of life-sustaining therapy in potentially salvageable patients [12]. Conversely, continuing aggressive treatment indefinitely for all non-wakeners places an immense emotional burden on families and strains limited medical resources. Thus, discriminating Late Awakeners from patients with irreversible vegetative states (Non-Awakeners) is a pressing clinical and ethical imperative.

The diagnostic dilemma is most acute in patients presenting in the diagnostic gray zone those who remain comatose (Glasgow Coma Scale Motor score [GCS-M] < 6) at 72 h but lack definitive hard signs of poor outcome, such as bilaterally absent N20 waves on somatosensory evoked potentials (SSEP) or severe cerebral edema on computed tomography (CT) [13]. In this indeterminate group, serum biomarkers like Neuron-Specific Enolase (NSE) are widely utilized. Although high NSE levels are strongly associated with poor outcomes, the interpretation of intermediate NSE elevations is fraught with uncertainty [14]. Furthermore, the kinetics of NSE release can be influenced by extracerebral factors, such as hemolysis, further complicating its diagnostic utility in this specific subgroup [15].

Despite the importance of this issue, there is a paucity of research specifically focused on the diagnostic characteristics of Late Awakeners. Most existing predictive models have focused on binary outcomes (Good vs. Poor) at a fixed time point, failing to capture the temporal trajectory of recovery in this gray-zone population. Identifying specific clinical or biochemical markers that can predict delayed but eventual recovery is essential for refining current prognostication protocols.

Therefore, the objective of this study was to investigate the prevalence of late awakening in a prospective cohort of OHCA survivors treated with TTM over an 11-year period and to identify diagnostic predictors that can distinguish Late Awakeners from Non-Awakeners among patients remaining comatose at 72 h. We hypothesized that Late Awakeners would exhibit a distinct diagnostic profile, characterized by intermediate biomarker levels and specific patterns of preserved brainstem reflexes, based on the following reasoning: first, Late Awakeners by definition recover consciousness, implying that their brain injury is potentially reversible rather than permanent; second, the brainstem, which governs fundamental arousal mechanisms, is relatively resistant to hypoxic injury compared to cortical structures, and therefore preserved brainstem reflexes may serve as a surrogate marker for recoverable arousal capacity; and third, NSE levels in an intermediate range may reflect a degree of neuronal injury that is compatible with eventual recovery, as opposed to the massive neuronal destruction indicated by very high NSE levels. Accordingly, a combination of these markers may provide objective evidence to support the extension of the observation period for this specific patient group.

## 2. Materials and Methods

### 2.1. Study Design and Subjects

This was a retrospective observational study conducted at a single tertiary academic medical center in Seoul, Republic of Korea. We analyzed data from a prospectively collected registry of adult (≥18 years) out-of-hospital cardiac arrest (OHCA) survivors treated with targeted temperature management (TTM). The study period spanned from January 2009 to December 2019. To minimize potential confounding effects related to the coronavirus disease 2019 (COVID-19) pandemic, such as changes in emergency medical service protocols or in-hospital isolation measures, patients admitted after January 2020 were excluded. The study protocol was approved by the Institutional Review Board (IRB) of Seoul St. Mary’s Hospital, the Catholic University of Korea (KC23RISI0264, approved 21 April 2023), and was conducted in accordance with the Declaration of Helsinki. As this was a retrospective observational study analyzing an existing clinical registry without any intervention, prospective registration in a clinical trial registry was not applicable. All patient data were de-identified prior to analysis; direct identifiers, including patient names and hospital registration numbers, were removed and replaced with sequential study identification numbers. The requirement for individual informed consent was waived by the IRB given the retrospective nature of the study and the use of de-identified data.

### 2.2. Study Population

During the study period, a total of 373 adult OHCA patients treated with TTM were screened. We specifically included patients who remained comatose, defined as failure to obey commands (Glasgow Coma Scale Motor score [GCS-M] < 6), at 72 h after the return of spontaneous circulation (ROSC). Patients were excluded if they: (1) died or had withdrawal of life-sustaining treatment (WLST) within 72 h; (2) regained consciousness (GCS-M = 6, i.e., obeying commands) within 72 h; or (3) had incomplete data on core prognostic markers (e.g., missing 72 h GCS or biomarker data). The final study cohort consisted of 213 patients. These patients were categorized into two groups based on the time of consciousness recovery: Late Awakeners, defined as those who first obeyed commands after 72 h from ROSC, and Non-Awakeners, defined as those who remained comatose until death or hospital discharge with a poor neurological outcome.

### 2.3. Targeted Temperature Management Protocol

All patients were managed according to a standardized post-cardiac arrest care protocol based on international guidelines. TTM was induced as soon as possible after ROSC using surface cooling devices (Arctic Sun^®^; BD, Franklin Lakes, NJ, USA) or intravascular cooling catheters (Coolgard^®^; ZOLL Medical, Chelmsford, MA, USA), depending on device availability and physician discretion. Core body temperature was continuously monitored through the integrated feedback system of each cooling device: for patients managed with the Arctic Sun^®^ surface cooling system, an esophageal temperature probe was used; for patients managed with the Coolgard^®^ intravascular cooling catheter, temperature was measured via the catheter’s integrated thermistor positioned in the inferior vena cava. Both methods provide reliable core temperature measurements consistent with current recommendations for temperature monitoring during TTM. The target temperature was maintained at either 33 °C or 36 °C for 24 h from the time the target was first achieved. Rewarming was initiated immediately upon completion of this 24 h maintenance phase without an intervening stabilization period and was performed at a controlled rate of 0.25 °C per hour until reaching 37 °C, which served as the operational endpoint for the active rewarming phase. This target was chosen as a practical protocol endpoint consistent with major TTM trials [2,3], after which temperature management was transitioned to fever prevention (maintenance of core temperature <37.8 °C for 72 h). We acknowledge that normothermia in clinical practice is generally defined as a range (approximately 36.5–37.5 °C); however, 37 °C was used as a standardized protocol-driven rewarming target across the study period. To control shivering and maintain TTM, sedation and analgesia were strictly managed. The primary sedative agent was midazolam, often combined with remifentanil or fentanyl for analgesia. Propofol was not used in our institution’s protocol during the study period. Neuromuscular blockade was routinely maintained in all patients (100%) during the cooling phase to prevent shivering, typically using continuous infusion or intermittent boluses of non-depolarizing agents (e.g., rocuronium or vecuronium). Sedatives were generally tapered or discontinued after rewarming to facilitate neurological examination, except in cases where continued sedation was necessary for seizure control or severe respiratory failure.

### 2.4. Data Collection and Prognostication

Neurological status was assessed serially at 0, 24, 48, and 72 h after ROSC. The key clinical parameters included the GCS score and brainstem reflexes (pupillary light reflex [PLR] and corneal reflex [CR]). Neurological examination including corneal reflex testing was performed at these standardized time points as part of our institutional TTM protocol. The time to obey commands was recorded in hours from ROSC for all patients who regained consciousness.

Serum Neuron-Specific Enolase (NSE) levels were measured at 0, 24, 48, and 72 h after ROSC. The maximum NSE value within the first 72 h (Max-NSE) was used for analysis. The measurement availability was as follows: NSE at 0 h (87.6%), 24 h (81.1%), 48 h (66.1%), and 72 h (40.7%); corneal reflex at 0 h (95.8%), 24 h (71.5%), 48 h (58.4%), and 72 h (55.6%). The decreased measurement rates at later time points reflect patient mortality and clinical prioritization. Renal function was monitored daily, and serum creatinine levels at admission and 72 h were recorded to evaluate potential metabolic confounding of sedative clearance. The primary outcome was late awakening, defined as first obeying commands (GCS-M = 6) after 72 h from ROSC. The secondary outcome was neurological status at 6-month follow-up, assessed using the Cerebral Performance Category (CPC) scale, with CPC 1–2 classified as good outcome and CPC 3–5 as poor outcome. Time interval variables were defined as follows: no-flow time, the interval from collapse to initiation of cardiopulmonary resuscitation (CPR); low-flow time, the interval from CPR initiation to ROSC; and total anoxic time, the sum of no-flow time and low-flow time.

Non-contrast brain computed tomography (CT) was performed immediately after ROSC in the emergency department to identify the cause of arrest and assess the extent of initial brain injury. The Grey-to-White Matter Ratio (GWR) was calculated as a quantitative measure of cerebral edema.

### 2.5. Statistical Analysis

Continuous variables were presented as medians with interquartile ranges (IQR) or means ± standard deviations, as appropriate, and compared using the Mann–Whitney U test or Student’s *t*-test. Categorical variables were expressed as frequencies and percentages and compared using the Chi-square test or Fisher’s exact test. To evaluate the diagnostic performance of NSE and other markers in distinguishing Late Awakeners from Non-Awakeners, Receiver Operating Characteristic (ROC) curve analysis was performed. The optimal cut-off value for NSE was determined using the Youden index. A multimodal diagnostic model was developed using multivariate logistic regression, incorporating variables that showed statistical significance in univariate analysis (*p* < 0.05) and had clinical plausibility, to identify independent predictors of late awakening. Model calibration was assessed using the Hosmer–Lemeshow goodness-of-fit test to evaluate the agreement between predicted probabilities and observed outcomes, and Brier score. Internal validation was performed using bootstrap resampling (1000 iterations) to estimate optimism-corrected AUC and assess potential overfitting. The events-per-variable (EPV) ratio was calculated to ensure adequate sample size for the logistic regression model. All statistical analyses were performed using SPSS version 26.0 and Python 3.10, and a *p*-value < 0.05 was considered statistically significant.

Missing data were handled using complete case analysis for the primary analysis. The proportion of missing data for key variables was: Max-NSE within 72 h (3.5%), corneal reflex at 72 h (44.4%), no-flow time (0.7%), and low-flow time (0.7%). The higher missing rate for 72-h corneal reflex testing reflects patient mortality before this time point. Patients with missing outcome data (time to obey commands) who died or had WLST before neurological recovery assessment were classified as Non-Awakeners based on their poor outcome trajectory. No imputation methods were employed given the nature of the outcome variable.

## 3. Results

### 3.1. Population

**Figure 1:**
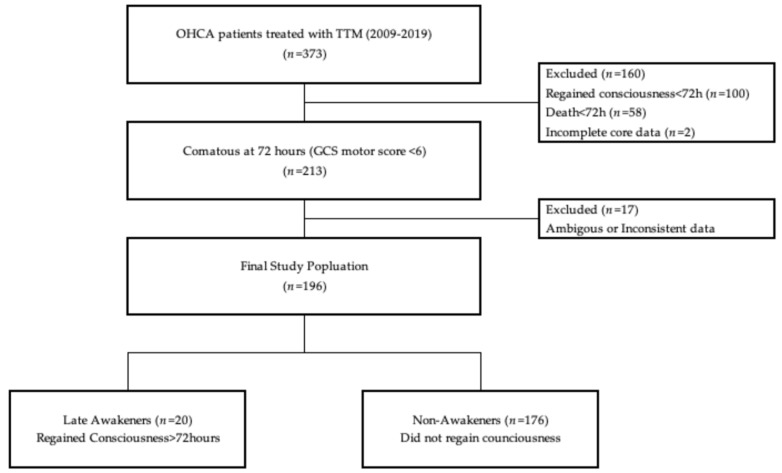
Study flowchart of the study population from 2009 to 2019.

During the study period, a total of 373 adult OHCA patients treated with TTM were screened. After excluding patients who regained consciousness within 72 h, died, or had incomplete data, 213 patients (57.1%) remained comatose (GCS motor score < 6) at 72 h post-ROSC and were included in the final analysis. Among this comatose cohort, 20 (9.4%) patients subsequently regained consciousness (Late Awakeners), while 176 (82.6%) remained comatose until death or hospital discharge with poor neurological outcomes (Non-Awakeners). Seventeen patients were excluded from the subgroup analysis due to ambiguous data. The selection process and study flow are illustrated in Figure 1.

The median time to obey commands in Late Awakeners was 105.6 h (IQR 81.2–199.8 h), equivalent to 4.4 days (IQR 3.4–8.3 days) from ROSC. When calculated from the time of normothermia achievement (median time to normothermia: 42.0 h), the median time from normothermia to awakening was 62.1 h (IQR 32.7–149.4 h), or approximately 2.6 days (IQR 1.4–6.2 days).

### 3.2. Baseline Characteristics

The baseline characteristics of Late Awakeners and Non-Awakeners are compared in Table 1. There were no significant differences between the two groups regarding age (*p* = 0.50), sex (*p* = 0.79), or the prevalence of comorbidities such as hypertension, diabetes, and chronic kidney disease. However, Late Awakeners exhibited significantly more favorable resuscitation features. They were more likely to present with a shockable rhythm (50.0% vs. 20.3%, *p* = 0.007) and had significantly shorter durations of no-flow time (median 1.0 vs. 5.0 min, *p* = 0.045) and total anoxic time (median 26.0 vs. 36.0 min, *p* = 0.007).

### 3.3. Neurological Status and Biomarkers at 72 h

We compared the neurological status and serum biomarker levels at 72 h between the two groups. The presence of brainstem reflexes at 72 h was significantly associated with late recovery. A preserved corneal reflex (CR) was observed in 85.0% (17/20) of Late Awakeners compared to only 20.2% of Non-Awakeners (*p* < 0.001).

**Figure 2:**
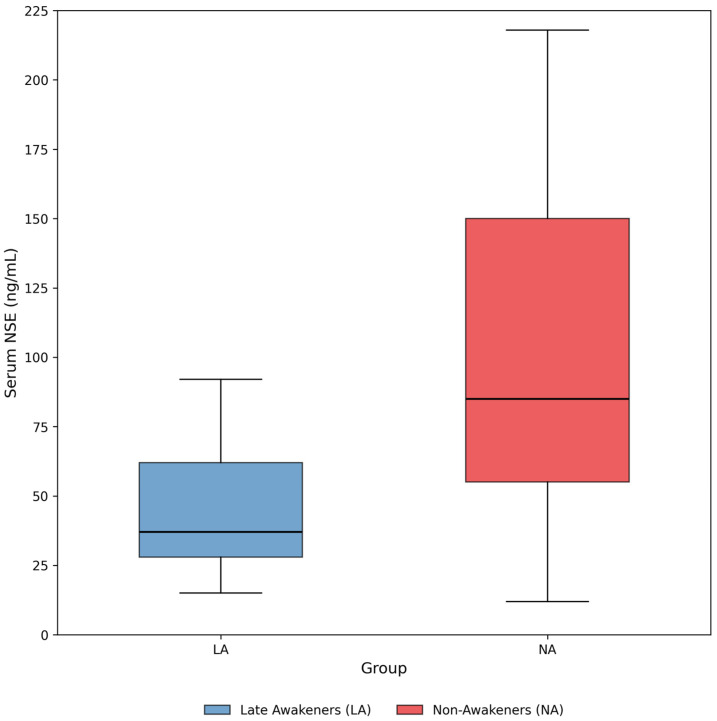
Distribution of maximum NSE levels within 72 h in Late Awakeners (LA) and Non-Awakeners (NA). The dashed line represents the cut-off value derived from ROC analysis.

The trajectory of serum NSE levels differed markedly between groups. As shown in Figure 2, Late Awakeners had significantly lower maximum NSE levels within the first 72 h (Median [IQR]: 33.0 [22.0–45.5] ng/mL) compared to Non-Awakeners (118.0 [55.0–280.0] ng/mL, *p* < 0.001).

**Figure 3:**
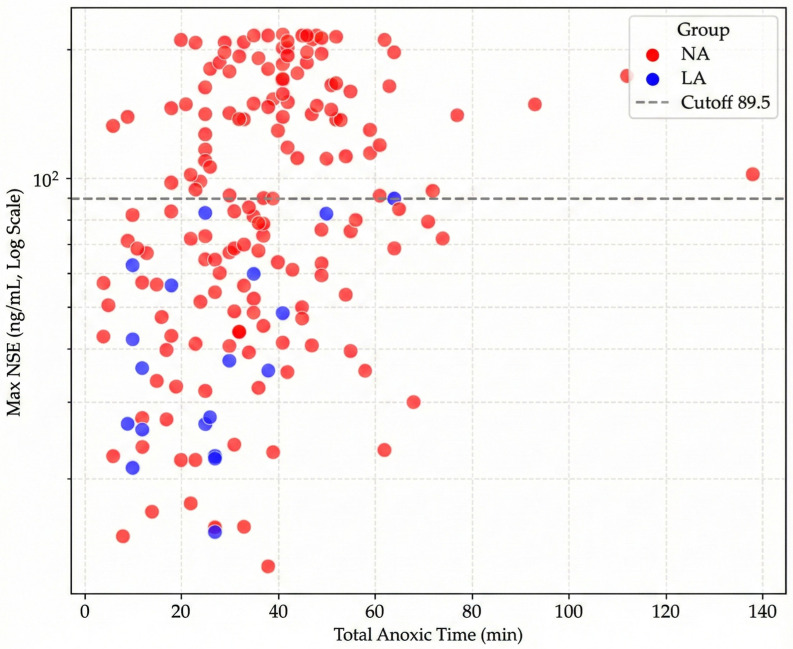
Scatter plot demonstrating the relationship between Total Anoxic Time (min) and Maximum NSE levels (ng/mL). Late Awakeners (Blue) are clustered in the region of lower anoxic time and lower NSE compared to Non-Awakeners (Red).

Furthermore, to visualize the relationship between ischemic burden and neuronal injury, we analyzed the correlation between total anoxic time and maximum NSE levels (Figure 3). Late Awakeners were distinctively clustered in the lower-left quadrant, characterized by shorter anoxic times and lower NSE levels, whereas Non-Awakeners showed a wide dispersion correlating with severe hypoxic injury.

### 3.4. Diagnostic Performance

We evaluated the diagnostic performance of NSE and clinical examination to discriminate Late Awakeners from Non-Awakeners in this gray zone population (Table 2).

First, ROC curve analysis identified an optimal Max-NSE cut-off value of 89.5 ng/mL (AUC 0.801, 95% CI 0.73–0.87). This cut-off yielded a high Sensitivity of 95.0% for predicting late awakening, but the Specificity was limited to 50.3%.

To improve diagnostic precision, we developed a multimodal model combining biochemical and clinical markers. The combination defined as Max-NSE < 90 ng/mL AND Preserved Corneal Reflex significantly improved the diagnostic performance. Although the sensitivity decreased to 70.0% compared to NSE alone, the Specificity increased markedly to 93.2%, effectively minimizing false positives. Furthermore, the AUC of the combined model, derived from logistic regression, was 0.899, demonstrating a superior overall discriminative ability compared to NSE alone (AUC 0.801).

Model calibration was assessed using the Hosmer–Lemeshow goodness-of-fit test. The multimodal model demonstrated good calibration (χ^2^ = 4.52, df = 8, *p* = 0.807), indicating no significant deviation between predicted and observed probabilities across risk strata. The Brier score was 0.066, further supporting adequate calibration.

Given the relatively small number of Late Awakeners (*n* = 20), internal validation was performed using 1000 bootstrap iterations to assess model stability. The optimism-corrected AUC was 0.896 (95% CI: 0.843–0.948), with minimal optimism of 0.003 (0.3% of apparent AUC), indicating negligible overfitting. The events-per-variable ratio was 10:1 (20 events/2 predictors), meeting the recommended minimum of 10:1 for logistic regression. Nevertheless, external validation in independent cohorts is warranted before clinical implementation.

### 3.5. Long-Term Outcomes of Late Awakeners

Among the Late Awakeners, neurological outcomes at hospital discharge were as follows: CPC 1 (good cerebral performance): 9 (45.0%), CPC 2 (moderate disability): 5 (25.0%), CPC 3 (severe disability): 5 (25.0%), and CPC 5 (death): 1 (5.0%). At 6-month follow-up (*n* = 19), 14 patients (73.7%) achieved good neurological outcome (CPC 1–2), with 12 (63.2%) achieving CPC 1. Thus, the majority of Late Awakeners achieved truly favorable outcomes rather than merely surviving with severe disability (Supplementary Table S1).

## 4. Discussion

This study investigated the diagnostic characteristics of patients who regain consciousness after the standard 72 h prognostication window. Our analysis of an 11-year prospective registry revealed that 9.4% of OHCA survivors who remained comatose at 72 h were Late Awakeners. These patients represented a distinct clinical phenotype characterized by favorable initial resuscitation features (shockable rhythm, short anoxic time), preserved brainstem reflexes (specifically corneal reflex), and serum NSE levels falling within an intermediate range (<90 ng/mL). We demonstrated that a multimodal diagnostic model combining Max-NSE < 90 ng/mL and preserved corneal reflex could discriminate Late Awakeners from Non-Awakeners with a high specificity of 93.2% and an AUC of 0.899. Importantly, internal validation using bootstrap resampling confirmed model stability with minimal overfitting (optimism 0.3%), and calibration analysis demonstrated good agreement between predicted and observed probabilities. This suggests that, for patients fitting this profile, the standard 72 h cut-off for prognostication is premature, and extended observation is warranted.

It is essential to distinguish between the phenomenon of late awakening and the achievement of long-term favorable neurological outcomes. Late awakening, defined as regaining consciousness after 72 h, does not automatically equate to meaningful functional recovery. In our cohort, while all Late Awakeners by definition regained consciousness, their ultimate outcomes varied considerably. At hospital discharge, 70.0% achieved good outcomes (CPC 1–2), while 25.0% had severe disability (CPC 3) and 5.0% died before discharge. Importantly, at 6-month follow-up, the proportion with good outcomes improved to 73.7%, suggesting that functional recovery may continue beyond hospital discharge in many patients. However, 26.3% of Late Awakeners remained with poor outcomes (CPC 3–5) at 6 months, indicating that late awakening alone should not be considered synonymous with favorable prognosis. Clinicians should counsel families that delayed awakening represents an opportunity for potential recovery, but outcomes remain uncertain and variable.

Based on our findings, we propose the following clinical recommendations for patients in the diagnostic gray zone (those meeting our multimodal criteria of Max-NSE < 90 ng/mL and preserved corneal reflex). First, we recommend extending the observation period to at least 5–7 days after normothermia, rather than adhering strictly to the 72-h rule. Our data showed that the median time to awakening was 2.6 days after normothermia, with an IQR extending to 6.2 days, suggesting that the majority of Late Awakeners will demonstrate signs of recovery within one week of normothermia. Second, during this extended observation period, serial neurological examinations should be performed daily, with particular attention to the return of brainstem reflexes and any signs of purposeful movement. Third, if no signs of neurological improvement are observed by 7–10 days post-normothermia, and additional investigations (such as MRI or EEG) confirm severe brain injury, WLST discussions may be appropriate at that time. However, we emphasize that these recommendations apply specifically to patients meeting our multimodal criteria; patients with clearly unfavorable prognostic markers (bilateral absent N20 on SSEP, NSE > 90 ng/mL, or absent brainstem reflexes) may not require such extended observation.

While our model focused on NSE and corneal reflex, several other prognostic modalities are commonly used in clinical practice and warrant discussion. Electroencephalography (EEG) provides valuable prognostic information, with highly malignant patterns (burst suppression with identical bursts, suppressed background) being strongly associated with poor outcomes. However, the interpretation of EEG patterns can be confounded by sedation, and the timing and method of EEG acquisition vary across centers. Continuous EEG monitoring, when available, may provide additional prognostic value, particularly for detecting subclinical seizures. Brain MRI with diffusion-weighted imaging (DWI) can detect cytotoxic edema as early as 24–48 h post-arrest, with extensive cortical and deep gray matter restriction predicting poor outcomes with high specificity. However, MRI is not always feasible in unstable ICU patients and requires transport from the ICU. Somatosensory evoked potentials (SSEP), specifically bilateral absence of the N20 cortical response, remain one of the most robust predictors of poor outcome with very high specificity. Unfortunately, systematic SSEP and MRI data were not available for our cohort, precluding formal comparison of these modalities with our NSE-corneal reflex model. Future prospective studies should evaluate the incremental value of combining our model with these complementary prognostic tools to further refine the identification of Late Awakeners

Current international guidelines recommend prognostication at 72 h post-ROSC to minimize the confounding effects of sedation [6,7]. However, our finding that approximately 1 in 10 comatose patients at this time point eventually recovers is consistent with previous literature. Paul et al. reported a late awakening rate of 12% in a multicenter cohort [7], highlighting that time to awakening varies significantly based on metabolic factors. Our data indicated that Late Awakeners had significantly shorter no-flow and total anoxic times compared to Non-Awakeners. This implies that Late Awakening in our cohort was not necessarily a result of severe brain injury recovering slowly, but rather moderate brain injury masked by other factors. In our institution, midazolam is the primary sedative agent used during TTM. Unlike propofol, midazolam has a context-sensitive half-life that increases with prolonged infusion and can accumulate in peripheral tissues, especially in the presence of hypothermia [8]. Therefore, the delayed recovery observed in our study likely reflects a combination of residual sedation and reversible metabolic suppression rather than irreversible structural damage.

Our observed late awakening rate of 9.4% warrants comparison with published data from other centers. Paul et al. reported a late awakening prevalence of 12% in a multicenter French cohort [7], while Gold et al. observed a rate of approximately 6.7% [16]. More recently, Rey et al. reported that 14.8% of initially comatose patients eventually achieved late neurological recovery [17]. This variability likely reflects differences in sedation practices, TTM protocols, institutional WLST policies, and the duration of the observation period before prognostic decisions are finalized. Notably, in regions with more conservative WLST approaches, the apparent rate of late awakening tends to be higher, as patients are afforded more time to demonstrate recovery [5].

The relationship between anoxic time and neurological outcomes has been well documented in the cardiac arrest literature. Sasson et al. demonstrated in a systematic review that each minute of delay to CPR initiation was associated with a 7–10% decrease in survival [18]. Similarly, Adrie et al. showed that prolonged no-flow and low-flow durations were independently associated with poor neurological outcomes [19]. Our finding that Late Awakeners had significantly shorter total anoxic times (median 26.0 vs. 36.0 min) aligns with this broader evidence base, suggesting that the severity of the initial ischemic insult is a critical determinant of recovery potential. In Late Awakeners, the relatively preserved ischemic burden may allow for neuronal recovery once confounding factors such as sedation and metabolic derangement are resolved.

An apparent paradox observed in our data warrants discussion: despite Late Awakeners having higher rates of witnessed arrest (80.0% vs. 61.6%) and bystander CPR (70.0% vs. 55.4%) compared to Non-Awakeners, these differences were not statistically significant (*p* = 0.142 and *p* = 0.31, respectively), whereas the differences in no-flow time were significant (median 1.0 vs. 5.0 min, *p* = 0.045). This discrepancy may reflect several factors. First, the quality of bystander CPR, rather than its mere presence, may be the critical determinant. Ineffective chest compressions—whether due to inadequate depth, rate, or interruptions—may have limited benefit despite timely initiation. Second, in some witnessed arrests, significant delays may occur between collapse recognition and CPR initiation due to bystander uncertainty or delayed emergency response activation. Third, documentation of ‘witnessed’ status may have been inconsistent across the registry period. These observations underscore the importance of high-quality CPR training and potentially the value of real-time CPR feedback devices.

A key contribution of this study is the identification of an NSE safe zone or upper limit for potential recovery. While high NSE is a well-established marker of poor outcome, the threshold for absolute non-recovery is often debated. In our study, the highest NSE value observed in a survivor who woke up late was 89.5 ng/mL. This creates a crucial Diagnostic Gray Zone between the normal range and this cut-off. Clinicians often face a dilemma when a patient has an NSE of 50 or 60 ng/mL levels that are elevated but not definitive for poor outcome. Our results suggest that in this intermediate range, the door for recovery remains open. Conversely, NSE levels exceeding 90 ng/mL were not observed among patients who subsequently achieved delayed awakening in this cohort and were associated with a very low likelihood of late neurological recovery (sensitivity 95% for non-awakening), suggesting that prolonged observation beyond this threshold is unlikely to provide additional clinical benefit.

Our NSE cut-off of 89.5 ng/mL can be contextualized within the broader landscape of published NSE thresholds. Stammet et al., in a substudy of the TTM trial, reported that NSE levels above 60 ng/mL at 48 h predicted poor outcome with high specificity [14]. The ERC/ESICM 2021 guidelines suggest that NSE values exceeding 60 ng/mL at 48 or 72 h, in combination with other unfavorable prognostic markers, should raise concern for poor outcome [4]. Some centers have adopted higher thresholds of 80–120 ng/mL for greater specificity [20]. Our findings are consistent with the concept of an intermediate NSE zone, where prognostic uncertainty is highest and clinical decision-making is most challenging. Within this zone—roughly between the lower threshold of clear recovery potential and the upper threshold of near-certain poor outcome—the addition of clinical examination findings, particularly brainstem reflexes, becomes essential for refining prognostic accuracy.

The Value of Multimodal Prognostication Our findings strongly advocate for a multimodal approach. Relying on NSE alone yielded a specificity of only ~50%, identifying too many false positives for potential recovery. However, adding the Corneal Reflex (CR) significantly sharpened the prediction. The brainstem is relatively resistant to hypoxic injury compared to the cortex; thus, a preserved CR indicates that the fundamental arousal centers are intact, even if cortical awareness is suppressed by sedation or edema [21]. Intermediate NSE levels combined with intact CR represent a meaningful signal favoring delayed prognostic assessment.

As shown in our logistic regression model, this combination achieved an AUC of 0.899, significantly outperforming biomarkers alone. If a patient at 72 h meets these criteria, we strongly advise against the immediate withdrawal of life-sustaining treatment (WLST). Instead, confirmatory tests such as brain MRI or delayed EEG should be prioritized, and the observation period should be extended to at least 5 to 7 days to allow for sedative clearance.

The discriminative performance of our combined model (AUC 0.899) compares favorably with published multimodal prognostication models. Sandroni et al. reported in a systematic review that multimodal approaches incorporating SSEP, EEG, and imaging achieved specificities ranging from 75% to 100%, depending on the combination of modalities used [13]. Notably, our model relies exclusively on bedside-available markers (serum NSE and corneal reflex testing) that require no specialized equipment or transport from the ICU, offering practical advantages in resource-limited settings. However, we acknowledge that the incorporation of additional modalities, particularly SSEP and brain MRI, could further refine prognostic accuracy. A recent study by Moseby-Knappe et al. demonstrated that combining biomarkers with imaging substantially improved the identification of patients with favorable prognosis [22]. Future prospective studies should evaluate whether integrating our NSE-corneal reflex model with these complementary modalities can further improve the identification of Late Awakeners in clinical practice.

The generalizability of our findings to different ICU practices requires consideration. Our institution’s TTM protocol utilized midazolam as the primary sedative, which has a longer context-sensitive half-time compared to propofol. Centers using propofol or other short-acting sedatives may observe different late awakening rates and timing patterns. Additionally, the specific NSE and corneal reflex thresholds identified may require local validation, particularly in centers with different laboratory assays or examination protocols. The multimodal approach itself, however—combining biomarker levels with clinical examination—represents a generalizable strategy that can be adapted to local practices.

This study has several limitations. First, as a retrospective analysis from a single tertiary center over an 11-year period, generalizability may be limited. TTM practices evolved during the study period, including changes in target temperature selection, though core protocol elements remained consistent. Second, our sedation protocol primarily utilized midazolam, which has prolonged context-sensitive half-time compared to propofol. Sedation protocols were not completely standardized, and cessation times were not systematically documented, preventing analysis of awakening relative to sedation discontinuation. The pharmacokinetics of sedatives can be altered by hypothermia and organ dysfunction, representing a potential confounding factor. Third, we did not incorporate quantitative neuroimaging or continuous EEG data, which could further refine prognostic accuracy. Additionally, CPC provides only a gross assessment of functional status; detailed quality of life assessments were not systematically collected. Fourth, self-fulfilling prophecy bias is inherent in retrospective prognostication studies. Clinicians’ prognostic expectations may influence WLST timing, and we cannot exclude that some Non-Awakeners might have awakened with extended observation. Finally, the Late Awakener group (*n* = 20) was relatively small. Despite adequate events-per-variable ratio and minimal overfitting on bootstrap validation, external validation is warranted before clinical implementation.

A significant proportion of cardiac arrest survivors who remain comatose at 72 h have the potential for late recovery. These Late Awakeners can be distinguished from patients with irreversible brain injury by a specific diagnostic profile: favorable resuscitation factors, preserved corneal reflexes, and NSE levels below 90 ng/mL. In this diagnostic gray zone, strict adherence to the 72 h rule may lead to the premature withdrawal of life-sustaining therapy. Clinicians should utilize this multimodal evidence to identify patients who deserve more time to wake up.

## 5. Conclusions

A substantial subset of cardiac arrest survivors who remain comatose at 72 h may still achieve meaningful late neurological recovery. Importantly, the majority of these Late Awakeners achieve truly favorable long-term outcomes, with 74.3% attaining good neurological function (CPC 1–2) at the six-month follow-up. These late awakeners can often be differentiated from patients with irreversible hypoxic-ischemic brain injury by a converging clinical profile, including favorable resuscitation characteristics, preserved corneal reflexes, and neuron-specific enolase levels below 90 ng/mL. In such patients, prognosis lies within a diagnostic gray zone. Rigid application of the 72 h rule in this context risks premature withdrawal of life-sustaining therapy. A multimodal assessment incorporating clinical, biochemical, and resuscitation-related factors—with demonstrated calibration and validated through bootstrap analysis—may therefore help identify patients for whom additional time for neurological recovery is warranted.

## Figures and Tables

**Table 1:** Baseline Characteristics of Late Awakeners and Non-Awakeners.

Characteristics	Late Awakeners (*n* = 20)	Non-Awakeners (*n* = 176)	*p*-Value
Demographics			
Age, median (IQR)	64.5 (50.8–67.5)	56.4 (45.3–70.0)	0.5
Male sex, *n* (%)	13 (65.0%)	125 (70.6%)	0.793
Comorbidities			
Hypertension	7 (35.0%)	62 (35.0%)	1
Diabetes Mellitus	7 (35.0%)	51 (28.8%)	0.752
Renal Disease (CKD)	3 (15.0%)	18 (10.2%)	0.454
Resuscitation Profiles			
Witnessed Arrest	16 (80.0%)	109 (61.6%)	0.142
Bystander CPR	14 (70.0%)	98 (55.4%)	0.31
Shockable Rhythm (VF/VT)	10 (50.0%)	36 (20.3%)	0.007 *
Cardiac Etiology	12 (60.0%)	80 (45.2%)	0.307
Time Intervals (min)			
No-flow time (Collapse to CPR)	1.0 (0.0–5.0)	5.0 (0.5–10.5)	0.045 *
Low-flow time (CPR to ROSC)	22.5 (11.5–27.5)	27.0 (18.0–37.0)	0.024 *
Total Anoxic time	26.0 (12.0–32.5)	36.0 (25.0–47.0)	0.007 *
TTM Target			
33 °C Target	17 (85.0%)	172 (97.2%)	0.036 *

Data are presented as median (IQR) for continuous variables and *n* (%) for categorical variables. * *p* < 0.05 indicates statistically significant difference.

**Table 2:** Diagnostic Performance of NSE, Corneal Reflex, and the Multimodal Model for Predicting Late Awakening.

Predictor	Cut-Off/Criteria	Sensitivity (95% CI)	Specificity (95% CI)	PPV	NPV	AUC
Single Marker						
Max NSE (0–72 h)	<89.5 ng/mL	95.00%	50.30%	17.80%	98.90%	0.801
Corneal Reflex	Present at 72 h	70.00%	88.70%	41.20%	96.30%	-
Multimodal						
Combined Model	NSE < 90 + CR (+)	70.00%	93.20%	53.80%	96.50%	0.899 *

The AUC for the Combined Model was derived from a logistic regression model incorporating both Max-NSE and Corneal Reflex as covariates. *: The AUC for the Combined Model was derived from a logistic regression model incorporating both Max-NSE and Corneal Reflex as covariates (optimism-corrected AUC: 0.896; 95% CI: 0.843–0.948).

## Data Availability

The data presented in this study are available on request from the corresponding author. The data are not publicly available due to legal restrictions.

## Supplementary Materials

The following supporting information can be downloaded at: https://www.mdpi.com/article/10.3390/diagnostics16040558/s1, Supplementary Materials Table S1. Neurological Outcomes of Late Awakeners at Hospital Discharge and 6-Month Follow-up.

## Abbreviations

The following abbreviations are used in this manuscript:

AKI	Acute Kidney Injury
AUC	Area Under the Curve
CI	Confidence Interval
CPC	Cerebral Performance Category
CR	Corneal Reflex
CT	Computed Tomography
GCS	Glasgow Coma Scale
HIBI	Hypoxic-Ischemic Brain Injury
IQR	Interquartile Range
LA	Late Awakeners
NA	Non-Awakeners
NPV	Negative Predictive Value
NSE	Neuron-Specific Enolase
OHCA	Out-of-Hospital Cardiac Arrest
PCAS	Post-Cardiac Arrest Syndrome
PLR	Pupillary Light Reflex
PPV	Positive Predictive Value
ROC	Receiver Operating Characteristic
ROSC	Return of Spontaneous Circulation
SSEP	Somatosensory Evoked Potential
TTM	Targeted Temperature Management
WLST	Withdrawal of Life-Sustaining Treatment
