# Supplementary material for: Differentiating Late Awakeners from Non-Awakeners in Comatose Cardiac Arrest Survivors: Diagnostic Value of Multimodal Monitoring in Patients with Indeterminate Prognosis
Source: Diagnostics (Basel). 2026 Feb 13;16(4):558. doi: 10.3390/diagnostics16040558 (PMC12939699; doi:10.3390/diagnostics16040558)
Supplement: Supplementary file 1 [file diagnostics-16-00558-s001.zip › diagnostics-4124059-supplementary.pdf]

Supplementary Table S1. Neurological Outcomes of Late Awakeners at Hospital Discharge and 6-Month Follow-up.

Outcome	Hospital Discharge (n = 20)	6-Month Follow-up (n = 19)
CPC 1 (Good cerebral performance)	9 (45.0%)	12 (63.2%)
CPC 2 (Moderate disability)	5 (25.0%)	2 (10.5%)
CPC 3 (Severe disability)	5 (25.0%)	3 (15.8%)
CPC 4 (Vegetative state)	0 (0%)	0 (0%)
CPC 5 (Death)	1 (5.0%)	2 (10.5%)
**Good outcome (CPC 1–2)**	**14 (70.0%)**	**14 (73.7%)**

CPC, Cerebral Performance Category. Good outcome was defined as CPC 1–2. One patient was lost to 6-month follow-up.
